# Comparative Analyses of Metabolomic Fingerprints and Cytotoxic Activities of Soft Corals from the Colombian Caribbean

**DOI:** 10.3390/md17010037

**Published:** 2019-01-09

**Authors:** Liliana Santacruz, Olivier P. Thomas, Carmenza Duque, Mónica Puyana, Edisson Tello

**Affiliations:** 1 Bioprospecting Research Group and Bioscience Doctoral Program, Faculty of Engineering, Campus Puente del Común, Universidad de La Sabana, 250001 Chía, Colombia; lilianasaci@unisabana.edu.co; 2 Marine Biodiscovery, School of Chemistry and Ryan Institute, National University of Ireland Galway (NUI Galway), University Road, H91 TK33 Galway, Ireland; 3 Departamento de Química, Universidad Nacional de Colombia, Carrera 30 # 45-03, 111321 Bogotá, Colombia; cduqueb@unal.edu.co; 4 Departamento de Ciencias Biológicas y Ambientales, Universidad Jorge Tadeo Lozano, Carrera 4 # 22-61, 110311 Bogotá, Colombia; monica.puyana@utadeo.edu.co

**Keywords:** metabolomics, soft-corals, *Pseudoplexaura flagellosa*, diterpenes, cytotoxic activity, LC-HRMSs

## Abstract

Soft corals (Cnidaria, Anthozoa, Octocorallia) are a diverse group of marine invertebrates that inhabit various marine environments in tropical and subtropical areas. Several species are recognized as prolific sources of compounds with a wide array of biological activities. Recent advances in analytical techniques, supported by robust statistical analyses, have allowed the analysis and characterization of the metabolome present in a single living organism. In this study, a liquid chromatography-high resolution mass spectrometry metabolomic approach was applied to analyze the metabolite composition of 28 soft corals present in the Caribbean coast of Colombia. Multivariate data analysis was used to correlate the chemical fingerprints of soft corals with their cytotoxic activity against tumor cell lines for anticancer purpose. Some diterpenoids were identified as specific markers to discriminate between cytotoxic and non-cytotoxic crude extracts of soft corals against tumor cell lines. In the models generated from the comparative analysis of PLS-DA for tumor lines, A549 and SiHa, the diterpene 13-keto-1,11-dolabell-3(E),7(E),12(18)-triene yielded a high score in the variable importance in projection. These results highlight the potential of metabolomic approaches towards the identification of cytotoxic agents against cancer of marine origin. This workflow can be useful in several studies, mainly those that are time consuming, such as traditional bioprospecting of marine natural products.

## 1. Introduction

Oceans cover around 71% of the Earth surface and host a great diversity of species, to which the marine environments have exerted a driving force, leading to new adaptive strategies and the synthesis of new metabolites [1]. Sessile and soft-bodied invertebrates, such as soft orals (Cnidaria, Anthozoa), have evolved particular metabolic pathways, leading to the production of chemical compounds as mechanisms of defense [2].

To date, seven compounds based on natural products of marine origin have been approved by the Food and Drug Administration (FDA) as pharmaceuticals. Among these, four have been specifically developed for the treatment of cancer: Cytarabine (Cytosar-U^®^, 1969 for the treatment of leukemia), eribulin mesylate (Halaven^®^, 2010 for the treatment of metastatic breast cancer), brentuximab vedotin (Adcetris^®^, 2011 for the treatment of anaplastic large T-cell lymphoma, and Hodgkin’s lymphoma), and trabectedin (Yondelis^®^, 2015 for the treatment of soft tissue sarcoma and ovarian cancer) [3]. Considering that cancer is still a major health concern worldwide [4], it is necessary to find new and effective cytotoxic agents against cancer. In this context, marine organisms are still largely unexplored and are promising sources of bioactive compounds with potential cytotoxic activity.

Chemical studies over the last 60 years have revealed that soft corals have developed an extraordinary ability to produce a large variety of compounds with unique chemical structures, usually associated with a broad range of biological activities [5]. Among some of the most interesting bioactive metabolites from soft corals are eleutherobin, originally isolated from the Australian soft coral, *Eleutherobia* sp., that exhibit anticancer properties [6] and the pseudopterosins from Caribbean *Antillogorgia elisabethae* that exhibit potent cytotoxic against five human cells lines (HeLa, PC-3, HCT116, MCF-7, and BJ), and anti-inflammatory and antimicrobial activity [7,8,9,10,11]. Due to the outstanding chemical diversity of soft corals, new tools that encompass the broad metabolome of a particular species belonging to this group will definitely open new opportunities to quickly target bioactive metabolites. Recent developments in analytical chemistry techniques, especially HPLC, MS, and NMR, have allowed the detection of thousands of metabolites with great sensitivity and specificity in a short time period [12]. Still, there is a prevailing question of whether metabolomic approaches could be used to quickly identify known or unknown metabolites as potential candidates for pharmaceutical applications [13].

Statistical analyses, such as multivariate data analyses, and particularly PCA (principal component analysis), group different samples through clustering or by determining outliers, whereas multivariate analysis methods allow the classification of the samples based on their cytotoxic activity against tumor cell lines. Thus, multivariate analyses can be used to correlate a set of metabolomic data to the results of a specific assay, making it possible to ascribe the metabolites that are likely involved in the detected cytotoxic activity against tumor cell lines [14].

The aim of this study was to establish a correlation between cytotoxic activity using three tumor lines, SiHa: Human cervical cancer, A549: Human lung adenocarcinoma, and PC3: Human prostatic carcinoma, and the chemical composition of 28 crude extracts from soft corals from the Colombian Caribbean. UPLC-HRMS (QToF) data were used to yield a metabolomic fingerprint of each crude extract based on terpenoids, [15,16], which are characteristic and well represented in these organisms and can be detected by the selected analytical method [17].

Orthogonal projections to latent structures discriminant analysis (OPLS-DA) was applied to the metabolomic data, considering that this analysis uses information in the Y matrix to decompose the X matrix into blocks of structured variation correlated and orthogonal to Y, respectively. In this investigation, OPLS-DA was used to discriminate the different extracts based on the metabolic fingerprints of all extracts and their cytotoxic activity against tumor cell lines, showing that extracts of *Eunicea clavigera* and *Pseudoplexaura flagellosa* were the extracts that mainly contributed to the separation. Additionally, partial least squares discriminant analysis (PLS-DA) was used to calculate the variable importance in projection (VIP) [18]. However, OPLS-DA can be used analogously to PLS-DA for discrimination, where the main benefit in interpretation using OPLS-DA compared to PLS-DA lies in the ability of OPLS-DA to separate predictive from non-predictive (orthogonal) variation [19]. Finally, the multivariate statistical analyzes established that a diterpenoid with a dolabellane type skeleton was responsible for the cytotoxic activity shown by the extracts against the SiHa and A549 cancer cell lines.

## 2. Results

### 2.1. Processing and Untargeted Data Acquisition

**Figure 1:**
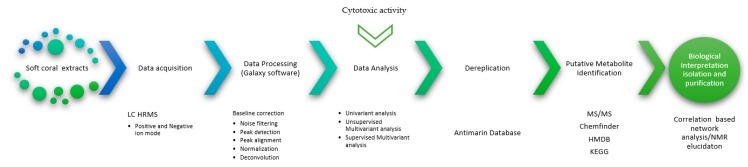
Metabolomic workflow pathway for 28 soft coral extracts. Data were processed with the Galaxy 4.0 software [20]. Metabolomic workflow included baseline correction, noise filtering, peak detection, peak alignment, normalization, deconvolution, and deisotoping.

A metabolomics workflow pathway was established for this research as is shown in Figure 1. In that way, a fingerprint metabolomics approach was applied to 28 soft coral extracts belonging to five different genera (*Plexaura*, *Antillogorgia*, *Eunicea*, *Plexaurella*, and *Pseudoplexaura*) by UPLC-HRMS in the positive ion mode, leading to 18,290 features analyzed with Galaxy 4.0 software [20]. The Script used for data processing is described in the Supplementary Table S3. The LC−MS data sets can be downloaded from the metabolomics repository, MetaboLights [21], with the reference study code, MTBLS777. Following this analysis, we established some quality criteria by which any variation of Quality Control (QC) samples around their mean (CVQC) > 30% was removed from the dataset; this reduced the matrix data from 18,290 to 12,060 features, representing a reduction of 34% of the total matrix.

Following our metabolomic workflow, three data matrices were generated. These data sets had the same number of features, but differed in the classification groups, following the in vitro cytotoxic activity of the soft coral extracts against the three cancer cell lines evaluated, human cervical cancer (SiHa), human lung adenocarcinoma (A549), and human prostatic carcinoma (PC3). L929 fibroblasts (L929; ATCC^®^CCL-1™) were used as the non-tumor cell line for toxicity control, however, the data obtained from L929 were not considered for the elaboration of the metabolomic matrix, due to the interest of this research.

**Figure 2:**
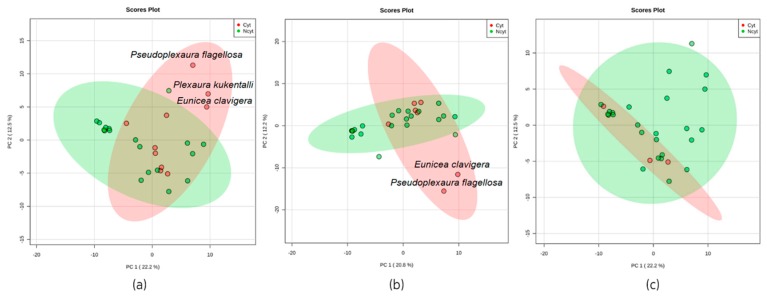
Principal component analysis score plot of metabolomics data from 28 extracts based on their cytotoxicity against three different cancer cell lines: (**a**) Human cervical cancer, SiHa, (**b**) human lung adenocarcinoma, A549, and (**c**) human prostatic carcinoma, PC3. Red dots indicate active extracts, and green dots represent extracts that were not active. An extract was considered active if it exhibited an inhibition of the tumor cell lines ≥50% at 20 μg/mL) [22]. The ellipses indicate confidence intervals of 95%.

**Figure 3:**
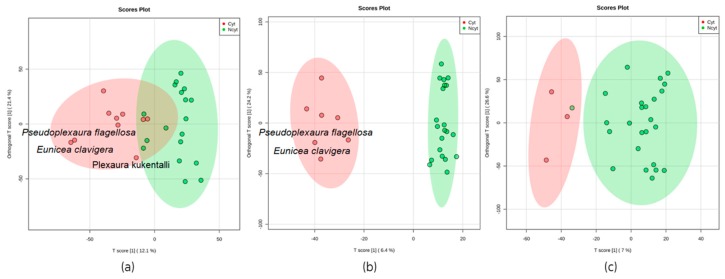
Supervised statistical analysis OPLS-DA score plots of metabolomic data of 28 soft coral extracts based on their cytotoxicity against three different cancer cell lines: (**a**) Human cervical cancer, SiHa, (**b**) human lung adenocarcinoma, A549, and (**c**) human prostatic carcinoma, PC3. Red dots indicate active extracts, and green dots represent extracts that were not active. An extract was considered active if it exhibited an inhibition of the tumor cell lines ≥ 50% at 20 μg/mL mL) [22]. The ellipses indicate confidence intervals of 95%.

For this research, it was established that an extract is considered active if it exhibited an inhibition of the tumor cell lines ≥50% at 20 μg/mL, following the guidelines of the National Cancer Institute (NCI) [22]. Supplementary Table S2 shows the results of the cytotoxicity presented by each extract against the tumor cell line, where the extract of the species, *Pseudoplexaura flagellosa* (code G17Ef), presented a value of 66.8% against SiHa and 71.5% against A549. The extract of the species, *Eunicea clavigera* (code C17Ec), presented a value of 61.3% against SiHa and 57.5% against A549. In agreement with Figure 2 and Figure 3 and using the multivariate statistical analysis, PCA and OPLS-DA, it was visualized that these two extracts presented greater separation of the group of extracts that presented cytotoxic activity for the tumor cell lines of A549 and SiHa.

### 2.2. Statistical Analysis

Using the data matrix (12,060 features), statistical analyses were run in the MetaboAnalyst platform [23]. PCA, orthogonal projection to latent structures discriminant analysis (OPLS-DA), and correlation analyses allowed the generation of variable importance in projection (VIP) scores. Figure 2 shows the results of the multivariate PCA considering their exposure to three different tumor lines, SiHa, A549, and PC3.

From the unsupervised statistical analysis method using PCA, it was possible to determine that both *Eunicea clavigera* and *Pseudoplexaura flagellosa* (Figure 2a,b) were the extract of the species that mainly contributed to the separation from others that did not exhibit cytotoxicity against the cell lines, SiHa and A549. Therefore, this analysis suggests that both extracts might share some metabolites responsible for the detected cytotoxicity. Additionally, it was also found that the extract of *Plexaura kuekenthali* (Figure 2a) was also included in the group of separate species because it exhibited cytotoxicity against the cell line, SiHa. The PCA analysis for the extract of this species that exhibited cytotoxicity against the PC3 cell line did not show a clear separation that allowed identification of the clusters or outliers responsible for the observed cytotoxicity as seen in Figure 2c.

For better separation between classes in the hyperspace plot and visualization of the features responsible for the discrimination between bioactive and non-bioactive extracts as cytotoxic against tumor cell lines, the statistical method, OPLS-DA, was performed (Figure 3). Regarding the OPLS-DA models observed in Figure 3b,c, a clear separation is observed between the extract of the species that presented cytotoxicity from those that did not present. Additionally, in Figure 3a,b, it can be observed that the extract of the species, *Pseudoplexaura flagellosa* and *Eunicea clavigera*, are closely related to each other again for the models using the SiHa and A549 tumor cell lines.

To infer statistically significant discrimination (*p*-value ≤ 0.05) between the two classes (cytotoxic and non-cytotoxic against tumor cell lines), a cross validation test was performed for the classification model with the three tumor cell lines, where the models were evaluated using both R2 and Q2 metrics. R2 values report the total amount of variance explained by the model in both the data (R2X) and independent variables (R2Y); the Q2 reports model accuracy and the ratio, Q2/R2, is a measure of cross-validation reproducibility, when the value is above 0.5 is considered with relevant associations [24]. The results showed values of Q2/R2 greater than 0.5 for the lines, A549 and SiHa, but not when the model was made with the tumor line of PC3 (see Table 1). These results show that for the first two cell lines, a good classification model was obtained, indicating the reliability of the models. Considering that the statistical analysis produced unreliable results for the model established with the PC3 tumor line, the subsequent analyzes will contemplate the data derived from the models when the lines of SiHa and A549 were used.

To complement the PLS-DA analyses, the molecular formula of the compounds was generated using the Agilent formula generator platform (MFG). Features that significantly contributed to clustering and discrimination were selected according to a threshold value of VIP ≥ 2.0 and a *p* value < 0.05 [25].

**Figure 4:**
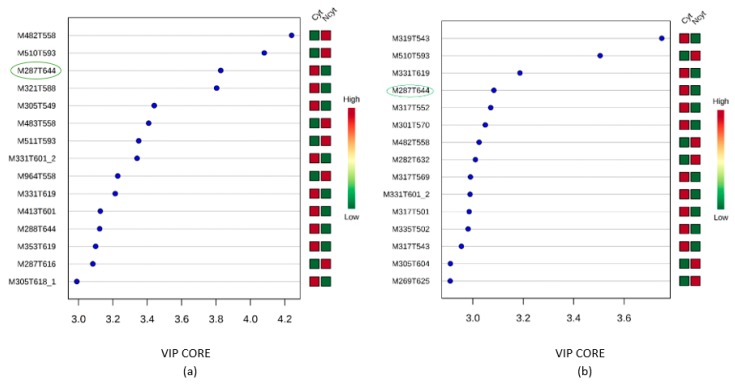
Results of the variable influence on projection (VIP) analyses to determine molecular features that contributed to extract clustering and discrimination in PLS-DA models against two cancer cell lines. Features were selected according to a threshold value of VIP ≥2.0 and a *p* value <0.05. The figures present the first 15 and most important features [26]. (**a**) SiHa; (**b**) A549.

According to the VIP analysis, 110 VIP were selected; Figure 4 shows the first 15 VIP for each cell line that mainly contributed to the separation of the extracts according to their cytotoxic potential.

As shown in Figure 4, the main feature that exhibited the greatest scores (SiHa score 3.1, A549 score 4.3) was M287T644, which was found in both models and seemed to be partly responsible for the separation. Other important features were M331T601 (SiHa score 3.0, A549 score 3.5) and M331T619 (SiHa score 3.2, A549 score 3.5).

### 2.3. Annotation, Dereplication, and Identification of the Feature M287T644

One of the great opportunities of metabolomics studies is the potential to identify new compounds by untargeted methods. A range of “dereplication” procedures are currently emerging to meet this challenge as key strategies for the identification of already known bioactive compounds and to improve the performance of natural product screening programs [27]. Here, we use a combinatorial approach for features selection with cytotoxic potential against tumor cell lines, using metabolomics analysis to establish the chemical profiles of soft coral extracts and dereplication using the AntiMarin^®^ data base to obtain a putative identification of the structures. By this way, we established that the characteristic, M287T644, corresponds to *m*/*z* of 287.2374 [M + H]^+^ consistent with the molecular formula, C_20_H_30_O, which was found in the extract of *Pseudoplexaura flagellosa*. Comparison with the database allowed us to propose two possible compounds for this molecular formula, eduenone [28] and dolabellatrienone [29].

**Figure 5:**
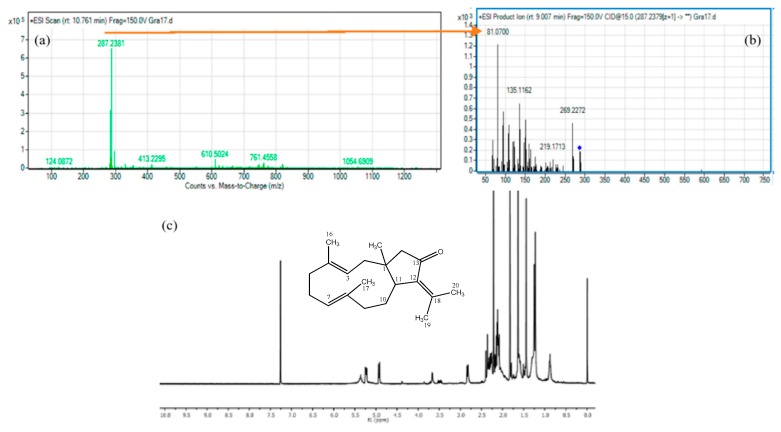
(**a**) MS spectrum of feature M287T644, isolated from the extract of *Pseudoplexaura flagellosa* (**b**) MS/MS spectrum of compound *m*/*z*: 287.2374 [M + H]^+^. (**c**). ^1^H NMR spectrum of 13-keto-1,11-dolabell-3(E),7(E),12(18)-triene and chemical structure of C_20_H_30_O.

To more precisely identify this feature, the MS/MS data of *m*/*z* 287.2374 [M + H]^+^ were recorded and analyzed using the MetFrag software [30]. The possibility that this feature corresponds to any of these two compounds, eduenone with a Diff of 0.59 ppm and dolabellatrienone with a Diff of 0.57 ppm, was confirmed. Considering that this feature was detected in the models made using the two tumor cell lines as one of the main features responsible for the separation of the group that presented cytotoxic activity, we decided to isolate the feature using repetitive HPLC-UV purifications. This compound was isolated as a yellowish oil with a molecular formula of C_20_H_30_O assigned based on HRESI-MS. The NMR spectroscopic features of this compound were indicative of a dolabellane compound similar to eduenone and dolabellatrienone. Some key ^1^H NMR signals allowed a quick identification of this compound: The chemical signals of the olefinic methine group in H-3 δ_H_ 5.24, dd, *J* = 11.4, 5.3 Hz, (eduenone: δ_H_ 6.30, br s; dolabellatriene: δ_H_ 5.24, dd, *J* = 11.2, 5.0 Hz), one cyclic-bearing methine in H-11 δ_H_ 2.83, d, *J* = 11.8 Hz, (eduenone: δ_H_ 2.99, br d, *J* = 12.0 Hz; dolabellatriene: δ_H_ 2.83, br d, *J* = 12.2 Hz), and one methyl group located in H-15 δ_H_ 1.23, s, (eduenone: δ_H_ 1.14, s; dolabellatriene: δ_H_ 1.23, s) were all consistent with the dolabellatriene. Therefore, based on the above results and the use of ^1^H-^1^H COSY and HMBC spectra, the structure of this compound was established as shown in Figure 5, a known dolabellane diterpenoid named 13-keto-1,11-dolabell-3(*E*),7(*E*),12(18)-triene previously isolated from *Eunicea calyculata* [31].

To evaluate the cytotoxicity of the compound, 13-keto-1,11-dolabell-3(E),7(E),12(18)-triene, two tumor cell lines, A549 and SiHa, were used in the MTT assay under five different concentrations, the dolabellatrienone exhibited values of IC_50_ = 0.02 µg/mL against A549 and IC_50_ = 0.03 µg/mL against SiHa.

## 3. Discussion

In the search for bioactive substances, marine organisms, such as soft corals, have led to metabolites with significant cytotoxic activities against different cancer cell lines [32]. In that way, this work established a metabolomic workflow pathway (Figure 2) that allowed correlation between the cytotoxic activities of 28 crude extracts from soft corals with their chemical composition. As seen in Figure 3, the statistical analysis, OPLS-DA, which seeks for maximal variance between the latent components, showed discrimination between the extracts that presented potential cytotoxicity against the extracts with lower cytotoxic potential. In addition, the data of the selected features were found to contribute to the separation of the extracts that presented cytotoxic potential and was found to be in common for the two models that presented a significant statistical difference (SiHa and A549), as shown in Figure 4. Considering this, it was decided to isolate the compound that corresponds to this feature from the extract identified as *Pseudoplexaura flagellosa*. This compound was identified by NMR and HRESI-MS analysis as 13-keto-1,11-dolabell-3(*E*),7(*E*),12(18)-triene C_20_H_30_O, *m*/*z* 287.2374 [M + H]^+^, a dolabellane compound previously isolated from *Eunicea calyculata* by Look and Fenical 1982 [31].

According with these metabolomics results, it is important to show some studies on compounds with dolabellane skeletons obtained from different marine organisms, which have shown cytotoxic potential against different tumor cell lines, scilicet: The compound, clavirolide G, isolated from the soft coral, *Clavularia viridis*, collected from the Xisha Islands in the South China Sea showed moderate cytotoxic activity against KB and HL-60 cells with IC_50_ values of 5.12 μg/mL and 5.92 μg/mL, respectively [33]. The compounds, clavinflols A and B, from the *Clavularia inflata* collected in Green Island, exhibited cytotoxicity against human oral epidermoid carcinoma (KB) cells (ED_50_ = 0.35 μg/mL) and showed selective activity towards human Hepa cells (ED_50_ = 1.2 µg/mL), respectively [34]. The compound, casearimene A, isolated from the species, *Casearia membranacea*, showed marginal activity against the tumor cell line, A549 (ED_50_ > 50 µg/mL) [35]. Also, five cytotoxic dolabellane diterpenes isolated in 2001 by Chang et al. from the Formosan soft coral, *Clavularia inflata*, showed moderate cytotoxicity against the A549 tumor cell line (ED_50_ = 7.74 to 50 μg/mL). Additionally, the compound, 7-hydroperoxydolabella-4(16),8(17),11(12)-triene-3,13-dione, was the most promising (ED_50_ = 0.57 μg/mL) [36]. The above studies demonstrated that diterpenes with a dolabellane skeleton have presented low and moderate cytotoxic activities against different tumor cell lines, as was presented against the tumor cell line, A549, which agrees with the results obtained in this research in which the compound, dolabellatrienone, exhibited values of IC_50_ = 0.02 μg/mL against A549, IC_50_ = 0.03 μg/mL against SiHa, and IC_50_ = 64.95 μg/mL against L929.

Finally, the main prospective application of this fingerprint metabolomic analysis in soft corals was aimed at the identification of potential metabolites with cytotoxic activity against cancer cell lines. This kind of metabolomic process can be useful in a several studies, mainly those that are time consuming, such as traditional bioprospecting of marine natural products; this takes around 1 to 3 years (depending on the sample and the metabolites), while the workflow established in this investigation took around four to six months to predict the presence and the identification of biologically active compounds (VIP) from soft coral extracts.

## 4. Materials and Methods

### 4.1. Materials

Solvents used for extraction, methanol, and dichloromethane were purchased from Merck (Darmstadt, Germany). For the chromatographic and spectrometric analysis, Acetonitrile, methanol, and formic acid of LC-MS were purchased from Sigma Aldrich (Dublin, Ireland). For use in cell culture, D-MEM (Dulbecco’s Modified Eagle Medium) (1X) and RPMI 1640 (Roswell Park Memorial Institute, Darmstadt, Germany were made by Gibco / Invitrogen, Paisley, UK. Fetal bovine serum (FBS), brand Eurobio (Les Ulis, France). Trypticase soy broth (TSB) and trypticase soy agar (TSA) brand Scharlau Co (Barcelona, Spain), PC3 cell line (prostate cancer), extracted from prostatic adenocarcinoma of a Caucasian man (ATCC^®^ CRL1435™), SiHa cancer cervical cell line (ATCC^®^ HTB-35™), and A549 cancer lung cell line (ATCC^®^ CCL-185™), and L929 fibroblast (ATCC^®^ CCL-1^TM^).

### 4.2. Methods

#### 4.2.1. Soft Coral Material Collection and Identification

Twenty-eight samples of soft corals (Supplementary Table S1) were collected at Santa Marta Bay, Colombia, in Punta Venado (N = 11° 16.26′ 87′′; W = 74° 12.24′ 58′′) at depths between 10 to 20 m. Small terminal fragments (of approximately 30 cm) were cut off the main soft coral colony with sharp scissors. Samples were air dried and then kept frozen until the moment of extraction at −80 °C.

Soft corals were identified by morphological and sclerite analyses [37,38,39]. For sclerite preparations, a small distal fragment of each sample was treated with 5% sodium hypochlorite. Once the organic matter was removed, sclerites were washed with distilled water and centrifuged at least four times. A final wash with ethanol followed by oven drying yielded sclerite preparations. Analysis of the sclerites was performed by microscopy [39,40]. Vouchers of all samples are stored at the Invertebrate collection of Instituto de Ciencias Naturales at Universidad Nacional de Colombia (Bogotá, Colombia) (Supplementary Table S1).

#### 4.2.2. General Experimental Procedures

UV measurements were obtained by the extraction of the diode array detector (DAD) signal in a PerkinElmer HPLC-DAD-ELSD FLEXAR LC(r) SYSTEM. High-resolution mass spectra (HRESIMS) were obtained with an Agilent 6540 mass spectrometer. Compound purification was carried out in a JASCO HPLC equipment, supplied with a PU4087 pump and a UV4070 UV/Vis detector using a preparative Phenyl-Hexyl OBD Column.

#### 4.2.3. Soft Coral Extraction, Sample Preparation, and UPLC/MS Analyses

The data acquisition was performed in an Agilent 6540 that generated a mass spectra zip file of 596 megabytes. Then, all data was preprocessed using the platform, Galaxy, which allows the automation of pipelines, ensuring reproducibility [20]. Afterwards, 1.0 g of dried powder from each soft coral was extracted at room temperature with a mixture of 1:1 DCM/MeOH, three times (30 mL) using an ultrasonic bath for 20 min. Debris were removed by centrifugation two times at 12,000× *g* for 5 min. Solvents were evaporated and dried extracts were passed through a C18 cartridge, eluting with MeOH to remove salts. Subsequently, the extracts were concentrated. The samples corresponding to the specie, *Pseudoplexaura flagellosa* (Gra 17) (one responsible for the separation according to OPLS-DA analysis), was purified by preparative RP-HPLC with a Phenyl-Hexyl OBD Column (XSelect CSH, 19 mm × 250 mm, 5 µm) and the optimization of gradient profiles was performed by selecting the mobile phase used for HPLC UV detection at λ 254 nm during 40 min. of acquisition time. Elution was done using water (A) and acetonitrile (B), both containing 0.1% formic acid with a gradient elution of 20%–50% B over 20 min, 50%–80% B over 10 min, 80%–100% B in 1 min, and holding for 5 min.

#### 4.2.4. Metabolomic Procedure

All samples used for metabolomics studies were analyzed using an Agilent 6540 mass spectrometer, which performs MS acquisition (*m*/*z* 300–3000) at 10 spectra/sec in high resolution mode at a resolution of 35,000 and mass accuracy of 1 ppm.

Electrospray Ionization (ESI) in positive and negative mode was used to ionize and detect compounds after chromatographic separations. General parameters of the MS1 mode source were gas flow of 12 L min^−1^, gas temperature of 300 °C, voltage charge of 2000 V, fragmentor of 150 V, capillary voltage of 3500 V, nebulizer pressure of 30 psi, and octopole RF Peak of 700 V.

The QC samples were analyzed intermittently during the analytical studies to assess the variance observed in the data. Chromatographic separation was achieved using a Phenyl-Hexyl HPLC column (150 mm × 3.0 mm, 1.9 µm Poroshell, Agilent Technologies, Santa Clara, CA, USA). The mobile phase consisted of (A) water with 0.1% formic acid to improve ionization and (B) methanol (MeOH) with 0.1% formic acid.

The UPLC injection volume on each run was 1.0 μL. The UPLC run contained blanks. QC samples and pooled samples [41] were intercalated throughout the UPLC run to control for any acquisition-dependent variation. Samples were filtered using a 0.2 µm Whatman^®^ membrane filter with a pore size of 0.2 μm (Merck, Germany) prior to injection.

Data were analyzed using the Agilent MassHunter Qualitative software (Version B.07.00). For formula generation, the Molecular Formula Generator algorithm (MFG) was used, which can automatically eliminate unlikely candidate compounds and rank the putative molecular formula according to their mass deviation, isotopic pattern accuracy, and elemental composition.

#### 4.2.5. Statistical Analyses

When analyzing large data sets from different samples, data variability can become a relevant complication: Hence, it might be to judge the quality of the data and assess their analytical variance. Hence, the use of a quality control (QC) allowed the minimization of this variability [42]. The QC samples contain the average of all metabolites within all the samples that were analyzed (0.1 µL of each octocoral extract was placed in a 1 mL vial), which were homogenized before the injection. QC samples were analyzed intermittently for the duration of the analytical study to assess the variance observed in the data throughout the sample preparation, data acquisition, and data pre-processing. Replicate injections should provide identical data for each injection, however, analytical variance is observed. Replicate QC injections can be used to measure this variance throughout the analytical study. We used a PCA to quickly assess the reproducibility of QC samples in an analytical run, and to determine the variance of the metabolite feature [43], pools were used and QC samples greater than 30% standard deviation were removed from the dataset [44]. An exploratory data analysis was first performed using PCA (Supplementary Figure S1). Additionally, the obtained signals from the blank were considered as interference and were subtracted (sn threshold = 3) [45].

For an untargeted approach, UPLC-MS chromatograms in negative and positive ion mode were pre-treated using an open source, platform independent software called Galaxy [20]. With this software, it was possible to exclude noise from LC-MS profiles (Noise level 5.0 E3, all data points below this intensity level were ignored). Parameters for data processing included the centwave method, 15 ppm, mzwid 0.015, and minfrac 0.3. After exporting the processed data in tabular format (.cvs file), further analysis of the data matrix was performed by MetaboAnalyst version 3.0 [23].

After PCA analyses, a PLS-DA (partial least square-discriminant analysis) and OPLS-DA (orthogonal partial least-squares-discriminant analysis) were performed with MetaboAnalyst software. OPLS-DA is a powerful tool for the analysis of qualitative data structures, while the prediction results are equivalent to classification using standard PLS-DA [19]. The PLS approach is a robust regression technique used for investigations of the relationship between two data sets. In this experiment, a discriminant classification was carried out using a PLS-DA based on the PLS algorithm, in which the discriminating variable was the cytotoxic activity. The combined application of PCA and OPLS-DA to spectral datasets yields valuable insights on both general spectral trends (PCA) and group-predictive spectral features (PLS) [46].

#### 4.2.6. Cytotoxicity Assays

Cell lines, human cervical cancer SiHa, human lung adenocarcinoma A549, human prostatic carcinoma PC3 and L929 fibroblasts, which was used as non-tumor cell line for toxicity control, were cultured in DMEM and RPMI 1640 media supplemented with 10% heat-inactivated fetal bovine serum (FBS) and penicillin-streptomycin (1%), at 37 °C in a 5% CO_2_ humidified atmosphere, until 100% confluence was achieved [47].

The in vitro cytotoxicity of the soft corals extracts was evaluated using the MTT method, which is a colorimetric assay based on the capacity of mitochondrial succinate dehydrogenase enzymes in live cells to reduce the yellow, water-soluble substrate, 3-(4,5-dimethyl thiazol-2-yl)-2,5-diphenyl tetrazolium bromide (MTT), into an insoluble, colored formazan product, which is measured spectrophotometrically following Mosmann [48] with modifications proposed by Denizot and Lang [49]. Cells were allowed to attach in 96-well plates (4.0 × 10^4^ cells/well) during 24 h. After 24 h, the medium was replaced with the extracts to be tested resuspended in DMSO (at a concentration of 2 mg/mL).

Soft coral extracts were evaluated at a concentration of 20 µg/mL. The greatest DMSO concentration was 0.1%, which was not cytotoxic to any of the cell lines. Prior to the assay, the supernatant was removed and 100 μL of 12 mM MTT solution in sterile PBS was added to each well and incubated at 37 °C for 4 h. The solution was removed, and extracts in dimethyl sulfoxide (DMSO) were added to each well, followed by incubation at 37 °C for 15 min. Optical density at 595 nm was read in an iMarkTM Microplate Reader. Cells cultured without extracts and doxorubicin were used as controls [50,51]. All tests were performed in triplicate. The viability percentage was calculated with Equation (1) and the cell inhibition was calculated with Equation (2) [32].

(1) $$\%viability=\frac{\left(Abs_{sample}\right)}{Abs_{control}}\times 100$$

where *Abs_sample_* is the absorbance of the cells treated with the test extract and *Abs_control_* is the absorbance of cells not treated with the test extract.

(2) %cell inhibition=100−Cell Survival

Extract toxicity is demonstrated by the inhibition of cell growth and division.

#### 4.2.7. Structure Elucidation

Fraction SP-9 obtained from the extract of *Pseudoplexaura flagellosa* (Gra17) was purified and then analyzed by LC–MS to establish the *m*/*z* of this purified fraction, yielding a (*m*/*z* of 287.2373). NMR spectra were acquired in an Agilent 600 MHz spectrometer equipped with a cryoprobe with pulse field gradient, and signals were referenced in ppm to the residual solvent signals (CDCl3 at 7.26 ppm, TMS at 0.00 ppm) (Table S3, NMR data of feature M287T644). 

## 5. Conclusions

In this work, metabolomics tools were successfully applied to compare the metabolomic profile of 28 extracts of soft corals. Data analyses using PCA, PLS-DA, and OPLS-DA were valuable to determine and highlight the potential cytotoxic metabolites from soft corals extracts. The Feature M287T644 was found as VIP in some extracts tested against SiHa and A549 cancer cell lines. Additionally, a dereplication process was important to putatively identify compounds suggested by statistical analyses as the main features explaining potential cytotoxicity in the tested extracts. The workflow established in this work led to the identification of the compounds 13-keto-1,11-dolabell-3(*E*),7(*E*),12(18)-triene as the main feature responsible for the separation of extracts with major cytotoxic potential, IC_50_ = 0.02 μg/mL against A549 and IC_50_ = 0.03 μg/mL against SiHa cancer cell lines, being in agreement with that reported in the literature for dolabellane type compounds. 

Additionally, as shown in Table S2, the G17 extract (*Pseudoplexaura flagellosa*) showed cytotoxic activity against the SiHa and A549 cell lines and was also cytotoxic against L929; however, the metabolite (VIP), C_20_H_30_O (13-keto-1,11-dolabell-3(E),7(E),12(18)-triene), isolated from this extract, which was the most responsible for the separation of the extracts of the soft corals that were classified as cytotoxic, did not reveal cytotoxicity against the normal cell line, L929, but presented moderate cytotoxicity against the cell line, A549.

The application of this fingerprint metabolomic analysis in soft corals was aimed at identifying potential metabolites with cytotoxic activity against tumor cell lines. This approach can be useful in several studies, like traditional bioprospecting of marine natural products, which are time consuming. In that way, the workflow established in this investigation allows the prediction of the presence of biologically active compounds from soft coral extracts in short periods of times.

## Figures and Tables

**Table 1:** Partial least squares-discriminant analysis (PLS-DA) parameters and permutation test for distinguishing between cytotoxic and non-cytototoxic groups from 28 extracts of soft corals tested against three cancer cell lines: SiHa: Human cervical cancer, A549: Human lung adenocarcinoma, PC3: Human prostatic carcinoma.

	PLS-DA Parameters		
Cell Lines	Q2	R2	Q2/R2
**SiHa**	0.43	0.67	0.64
**A549**	0.34	0.65	0.52
**PC3**	−0.23	0.78	−0.29

Parameters based on Q2 indicate the best classifier of PLS-DA analyses using a 10-fold cross-validation method. PLS-DA: partial least squares-discriminant analysis, Q2: predictive capability, R2: correlation coefficients.

## Supplementary Materials

The following are available online at http://www.mdpi.com/1660-3397/17/1/37/s1, Figure S1: Principal component analysis (PCA); in the triangle the pools (QC) are shown. Table S1: Name of species of soft corals used in this research a Collection Code (ICN), assigned by Collection of the Institute of Natural Sciences of the National University of Colombia, (Bogotá, Colombia). Table S2: Percentage of cytotoxic activity against three cancerous cell lines. (1 if it is above 50% 0 if it is below). Table S3: Scrip using Galaxy software to UPLC-MS data extraction.

## References

[1] Blunt J., Copp B., Keyzers R., Munro M., Prinsep M. (2012). Marine natural products. Nat. Prod. Rep..

[2] Jiménez J., Marfil A., Francesch C., Cuevas M., Alvarez A., Albericio F. (2007). Productos naturales de origen marino. Investig. Cienc..

[3] Dyshlovoy S., Honecker F. (2018). Marine Compounds and Cancer: 2017 Updates. Mar. Drugs.

[4] Ruiz Torres V., Encinar J.A., Herranz López M., Pérez Sánchez A., Galiano V., Barrajón Catalán E., Micol V. (2017). An updated review on marine anticancer compounds: The use of virtual screening for the discovery of small-molecule cancer drugs. Molecules.

[5] Bennett D. (2005). Growing Pains for Metabolomics. Scientist.

[6] Ojima I., Chakravarty S., Inoue T., Lin S., He L., Horwitz S.B., Kuduk S.D., Danishefsky S.J. (1999). A common pharmacophore for cytotoxic natural products that stabilize microtubules. Proc. Natl. Acad. Sci. USA.

[7] Correa H., Valenzuela A.L., Ospina L.F., Duque C. (2009). Anti-inflammatory effects of the gorgonian Pseudopterogorgia elisabethae collected at the Islands of Providencia and San Andrés (SW Caribbean). J. Inflamm..

[8] Correa H., Aristizabal F., Duque C., Kerr R. (2011). Cytotoxic and antimicrobial activity of pseudopterosins and seco-pseudopterosins isolated from the octocoral Pseudopterogorgia elisabethae of San Andrés and Providencia Islands (Southwest Caribbean Sea). Mar. Drugs.

[9] Correa H. (2012). Estudios de Bioprospección del coral blando Pseudopterogorgia Elisabethae como Fuente de Sustancias con Actividad Biológica Fase IV. Ph.D. Thesis.

[10] Marrero J., Rodríguez A.D., Baran P., Raptis R.G. (2004). Ciereszkolide: Isolation and structure characterization of a novel rearranged cembrane from the caribbean sea plume *Pseudopterogorgia kallos*. Eur. J. Org. Chem..

[11] Look S.A., Fenical W., Jacobst R.S., Clardyt J.O.N. (1986). The pseudopterosins: Anti-inflammatory and analgesic natural products from the sea whip *Pseudopterogorgia elisabethae*. Proc. Natl. Acad. Sci. USA.

[12] Deng L., Gu H., Zhu J., Nagana Gowda G.A., Djukovic D., Chiorean E.G., Raftery D. (2016). Combining NMR and LC/MS using backward variable elimination: Metabolomics analysis of colorectal cancer, polyps, and healthy controls. Anal. Chem..

[13] Lindon J.C., Holmes E., Bollard M.E., Stanley E.G., Nicholson J.K. (2004). Metabonomics technologies and their applications in physiological monitoring, drug safety assessment and disease diagnosis. Biomarkers.

[14] Tistaert C., Chataigné G., Dejaegher B., Rivière C., Hoai N.N., Van M.C., Quetin-leclercq J., Heyden Y. (2012). Vander Multivariate data analysis to evaluate the fingerprint peaks responsible for the cytotoxic activity of *Mallotus* species. J. Chromatogr. B.

[15] Leal M.C., Madeira C., Brandão C.A., Puga J., Calado R. (2012). Bioprospecting of marine invertebrates for new natural products—A chemical and zoogeographical perspective. Molecules.

[16] Wei X., Rodríguez A.D., Baran P., Raptis R.G. (2010). Dolabellane-type diterpenoids with antiprotozoan activity from a southwestern Caribbean gorgonian octocoral of the genus *Eunicea*. J. Nat. Prod..

[17] Maille G., Qin C., Siuzdak G. (2006). Nonlinear Data Alignment for UPLC—MS and HPLC—MS Based Metabolomics: Quantitative Analysis of Endogenous and Exogenous Metabolites in Human Serum. Anal. Chem..

[18] Szymanska E., Saccenti E., Smilde A.K., Westerhuis J.A. (2012). Double-check: Validation of diagnostic statistics for PLS-DA models in metabolomics studies. Metabolomics.

[19] Rantalainen M., Cloarec O., Nicholson J.K., Holmes E., Trygg J. (2006). OPLS discriminant analysis: Combining the strengths of PLS-DA and SIMCA classification. J. Chemiometr..

[20] Goecks J., Nekrutenko A., Taylor J., Afgan E., Ananda G., Baker D., Blankenberg D., Chakrabarty R., Coraor N., Goecks J. (2010). Galaxy: A comprehensive approach for supporting accessible, reproducible and transparent computational research in the life sciences. Genome Biol..

[21] Haug K., Salek R.M., Conesa P., Hastings J., De Matos P., Rijnbeek M., Mahendraker T., Williams M., Neumann S., Rocca-Serra P. (2013). MetaboLights—An open-access general-purpose repository for metabolomics studies and associated meta-data. Nucleic Acids Res..

[22] Hostettman K. (1991). Methods in Plant Biochemistry. Assays for Bioactivity.

[23] Xia J., Psychogios N., Young N., Wishart D.S. (2009). MetaboAnalyst: A web server for metabolomic data analysis and interpretation. Nucleic Acids Res..

[24] Moltu S.J., Sachse D., Blakstad E.W., Strømmen K., Nakstad B., Almaas A.N., Westerberg A.C., Rønnestad A., Brække K., Veierød M.B. (2014). Urinary metabolite profiles in premature infants show early postnatal metabolic adaptation and maturation. Nutrients.

[25] Chiu C.Y., Yeh K.W., Lin G., Chiang M.H., Yang S.C., Chao W.J., Yao T.C., Tsai M.H., Hua M.C., Liao S.L. (2016). Metabolomics reveals dynamic metabolic changes associated with age in early childhood. PLoS ONE.

[26] Xia J., Sinelnikov I.V., Han B., Wishart D.S. (2015). MetaboAnalyst 3.0—Making metabolomics more meaningful. Nucleic Acids Res..

[27] Hubert J., Nuzillard J.M., Renault J.H. (2017). Dereplication strategies in natural product research: How many tools and methodologies behind the same concept?. Phytochem. Rev..

[28] National Center for Biotechnology Information Eduenone. https://pubchem.ncbi.nlm.nih.gov/compound/10424127.

[29] National Center for Biotechnology Information Dolabellatrienone. https://pubchem.ncbi.nlm.nih.gov/compound/10469260.

[30] Wolf S., Schmidt S., Müller Hannemann M., Neumann S. (2010). In silico fragmentation for computer assisted identification of metabolite mass spectra. BMC Bioinform..

[31] Look S.A., Fenical W. (1982). New Bicyclic Diterpenoids from the Caribbean Gorgonian Octocoral Eunicea calyculata. J. Org. Chem..

[32] Patel S., Gheewala N., Suthar A., Shah A. (2009). In-Vitro cytotoxicity activity of Solanum Nigrum extract against Hela cell line and Vero cell line. Int. J. Pharm. Pharm. Sci..

[33] Gao Y., Xiao W., Liu H.C., Wang J.R., Yao L.G., Ouyang P.K., Wang D.C., Guo Y.W. (2017). Clavirolide G, a new rare dolabellane-type diterpenoid from the Xisha soft coral *Clavularia viridis*. Chin. Chem. Lett..

[34] Shen Y.C., Pan Y.L., Ko C.L., Kuo Y.H., Chen C.Y. (2003). New dolabellanes from the Taiwanese soft coral clavularia inflata. J. Chin. Chem. Soc..

[35] Chang K.C., Duh C.Y., Chen I.S., Tsai I.L. (2003). A cytotoxic butenolide, two new dolabellane diterpenoids, a chroman and a benzoquinol derivative formosan *Casearia membranacea*. Planta Med..

[36] Duh C.Y., Chia M.C., Wang S.K., Chen H.J., El-Gamal A.A.H., Dai C.F. (2001). Cytotoxic dolabellane diterpenes from the Formosan soft coral *Clavularia inflata*. J. Nat. Prod..

[37] Frederickm M. (1988). Bayer the Shallow-Water Octocorallia of the West Indian Region: A Manual for Marine Biologists.

[38] Sánchez J.A., Wirshing H.H. (2005). A field key to the identification of tropical western Atlantic zooxanthellate octocorals (Octocorallia: Cnidaria). Caribb. J. Sci..

[39] Sánchez J.A. (1998). Sistemática Filogenética del Género Eunicea Lamouroux, 1816 (Octocorallia: Gorgonacea: Plexauridae) con Aspectos Sobre la Historia Natural de Algunas Especies en el Caribe Colombiano. Master’s Thesis.

[40] Sánchez J.A., Lasker H.R. (2003). Patterns of morphological integration in marine modular organisms: Supra-module organization in branching octocoral colonies. R. Soc..

[41] Sangster T., Major H., Plumb R., Wilson A., Wilson I. (2006). A pragmatic and readily implemented quality control strategy for HPLC-MS and GC-MS-based metabonomic analysis. Analyst.

[42] Godzien J., Alonso-Herranz V., Barbas C., Armitage E.G. (2015). Controlling the quality of metabolomics data: New strategies to get the best out of the QC sample. Metabolomics.

[43] Dunn W., Broadhurst D., Edison A., Guillou C., Viant M., Bearden D., Beger R. (2017). Quality assurance and quality control processes: Summary of a metabolomics community questionnaire. Metabolomics.

[44] Gorrochategui E., Jaumot J., Lacorte S., Tauler R. (2016). Data analysis strategies for targeted and untargeted LC-MS metabolomic studies: Overview and workflow. TrAC Trends Anal. Chem..

[45] Brown M., Dunn W.B., Dobson P., Patel Y., Winder C.L., Francis-McIntyre S., Begley P., Carroll K., Broadhurst D., Tseng A. (2009). Mass spectrometry tools and metabolite-specific databases for molecular identification in metabolomics. Analyst.

[46] Gromski P.S., Muhamadali H., Ellis D.I., Xu Y., Correa E., Turner M.L., Goodacre R. (2015). A tutorial review: Metabolomics and partial least squares-discriminant análisis—A marriage of convenience or a shotgun wedding. Anal. Chim. Acta.

[47] Iwagawa T., Hashimoto K., Yokogawa Y., Okamura H., Nakatani M., Doe M., Morimoto Y., Takemura K. (2009). Cytotoxic biscembranes from the soft coral *Sarcophyton glaucum*. J. Nat. Prod..

[48] Mosmann T. (1983). Rapid colorimetric assay for cellular growth and survival: Application to proliferation and cytotoxicity assays. J. Immunol. Methods.

[49] Denizot F., Lang R. (1986). Rapid colorimetric assay for cell growth and survival. J. Immunol. Methods.

[50] Al-Ghamdi S.S. (2008). Time and dose dependent study of doxorubicin induced DU-145 cytotoxicity. Drug Metab. Lett..

[51] Shaikh K.S., Pawar A., Aphale S.R., Moghe A.S. (2012). Effect of vesicular encapsulation on in-vitro cytotoxicity of ciclopirox olamine. Int. J. Drug Deliv..

