# Supplementary material for: Comparative Analyses of Metabolomic Fingerprints and Cytotoxic Activities of Soft Corals from the Colombian Caribbean
Source: Mar Drugs. 2019 Jan 9;17(1):37. doi: 10.3390/md17010037 (PMC6356725; doi:10.3390/md17010037)
Supplement: Supplementary file 1 [file marinedrugs-17-00037-s001.pdf]

marine drugs

Supplementary Information

Comparative Analyses of Metabolomic Fingerprints and Cytotoxic Activities of Soft Corals from the Colombian Caribbean

Liliana Santacruz [1], Olivier P. Thomas [2,*], Carmenza Duque [3], Mónica Puyana [4] and Edisson Tello [1,*]

[1] Bioprospecting Research Group and Bioscience Doctoral Program, Faculty of Engineering, Campus Puente del Común, Universidad de La Sabana, Chía, Colombia
[2] Marine Biodiscovery, School of Chemistry and Ryan Institute, National University of Ireland Galway (NUI Galway), University Road, H91 TK33 Galway, Ireland
[3] Departamento de Química, Universidad Nacional de Colombia, Carrera 30 # 45-03, Bogotá, Colombia; cduqueb@unal.edu.co
[4] Departamento de Ciencias Biológicas y Ambientales, Universidad Jorge Tadeo Lozano, Carrera 4 # 22-61, Bogotá, Colombia; monica.puyana@utadeo.edu.co
* Correspondence: olivier.thomas@nuigalway.ie (L.S.); edisson.tello@unisabana.edu.co (E.T.); Tel.: +353-(0)9-149-3563 (L.S.); Tel.: +57-32-0699-5696 (E.T.)

Index of Figures

Figure S1. Principal component analysis (PCA); in the triangle the pools (QC) are shown. 3

Index of Tables

Table S1. Name of species of soft corals used in this research a Collection Code (ICN), assigned by Collection of the Institute of Natural Sciences of the National University of Colombia, (Bogotá, Colombia). 2

Table S2. Percentage of cytotoxic activity against three cancerous cell lines. (1 if it is above 50% 0 if it is below) 4

Table S3. Scrip using Galaxy software to UPLC-MS data extraction................ 5

Table 1. Name of species of soft corals used in this research a Collection Code (ICN), assigned by Collection of the Institute of Natural Sciences of the National University of Colombia, (Bogotá, Colombia).

Name Assigned	Name of the Species	Accession	Code ICN-UN
Cali 2	*Plexaura kukenthali*	C2P	ICN-MHN (Po)-CO-271
Cali 4	*Pseudopterogorgia albatrossae*	C4P	ICN-MHN (Po)-CO-272
Cali 6	*Eunicea succinea*	C6E	ICN-MHN (Po)-CO-273
Cali 8	*Eunicea succinea plantaginea*	C8E	ICN-MHN (Po)-CO-274
Cali 9	*Eunicea clavigera*	C9E	ICN-MHN (Po)-CO-275
Cali 11	*Eunicea flexuosa*	C11E	ICN-MHN (Po)-CO-276
Cali 12	*Plexaura cf. nina*	C12P	ICN-MHN (Po)-CO-279
Cali 13	*Eunicea fusca*	C13E	ICN-MHN (Po)-CO-277
Cali 15	*Pseudopterogorgia albatrossae*	C15Pst	ICN-MHN (Po)-CO-278
Cali 16	*Eunicea fusca*	C16E	ICN-MHN (Po)-CO-277
Cali 17	*Eunicea clavigera thin form*	C17E	ICN-MHN (Po)-CO-280
Cali 19	*Eunicea flexuosa*	C19E	ICN-MHN (Po)-CO-276
Cali 24	*Plexaurella nutans*	C24Plr	ICN-MHN (Po)-CO-281
Gra 1	*Plexaurella fusifera*	G1P Plr	ICN-MHN (Po)-CO-282
Gra 2	*Plexaura homomalla*	G2P	ICN-MHN (Po)-CO-283
Gra 3	*Eunicea clavigera thin form*	G3E	ICN-MHN (Po)-CO-284
Gra 4	*Eunicea asperula*	G4E	ICN-MHN (Po)-CO-285
Gra 5	*Eunicea clavigera*	G5E	ICN-MHN (Po)-CO-275
Gra 6	*Plexaura kukenthali*	G6P	ICN-MHN (Po)-CO-292
Gra 8	*Eunicea clavigera*	G8E	ICN-MHN (Po)-CO-275
Gra 9	*Plexaura sp.*	G9P	ICN-MHN (Po)-CO-286
Gra 10	*Eunicea knightii*	G10E	ICN-MHN (Po)-CO-287
Gra 13	*Eunicea clavigera*	G13E	ICN-MHN (Po)-CO-275
Gra 14	*Eunicea cf. calyculata*	G14E	ICN-MHN (Po)-CO-288
Gra 17	*Pseudoplexaura flagellosa*	G17Ps	ICN-MHN (Po)-CO-289
Gra 18	*Plexaura kukenthali*	G18P	ICN-MHN (Po)-CO-292
Gra 19	*Eunicea tayrona*	G19E	ICN-MHN (Po)-CO-290
Gra 22	*Plexaurella sp.*	G22P	ICN-MHN (Po)-CO-291

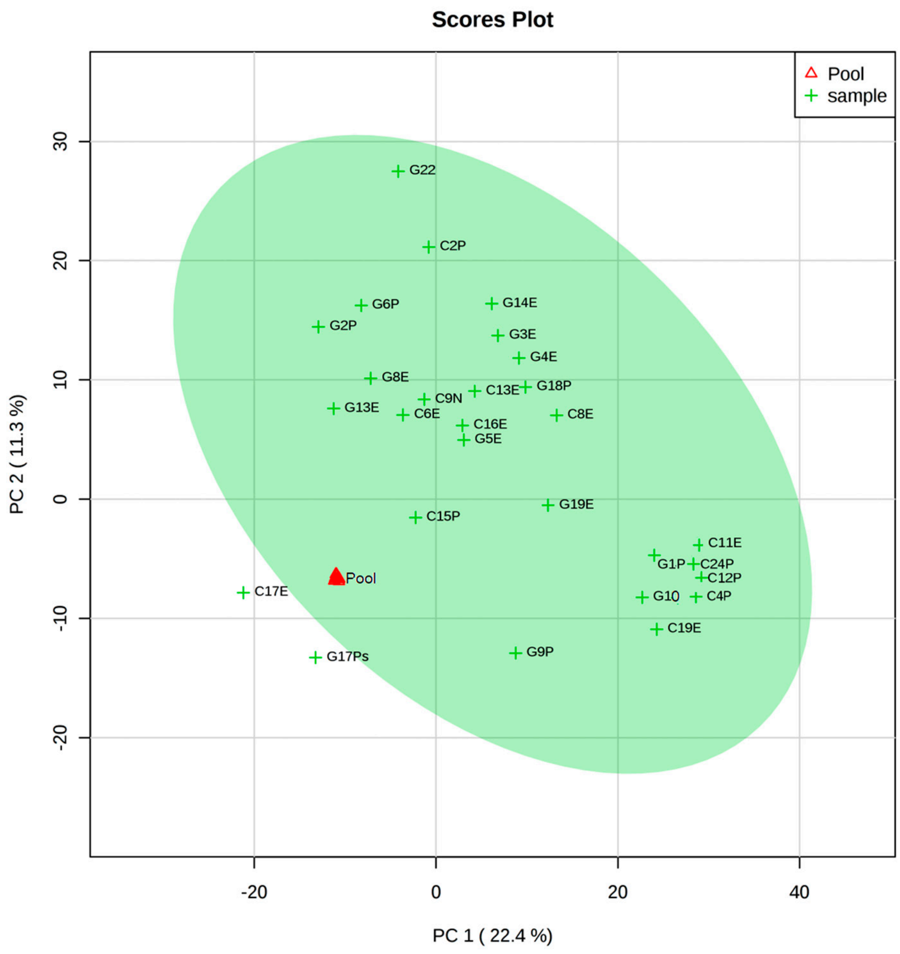

Figure 1. Principal component analysis (PCA); in the triangle the pools (QC) are shown.

Table 2. Percentage of cytotoxic activity against three cancerous cell lines. (1 if it is above 50% 0 if it is below)**.**

Obs	Name	SiHa (%)	PC3 (%)	A549 (%)	L929 (%)	SiHa	PC3	A549
**1**	G9Px	84.5	20.4	31.0	49.8	1	0	0
**2**	C6Es	69.0	61.7	35.1	28.9	1	1	0
**3**	G17Ef	66.8	40.3	71.5	65.9	1	0	1
**4**	G14Ec	63.5	22.0	65.7	19.2	1	0	1
**5**	C17Ec	61.3	17.0	27.5	13.8	1	0	0
**6**	C16Ee	60.5	30.0	39.1	21.3	1	0	0
**7**	G3Ec	59.9	33.1	45.3	5.8	1	0	0
**8**	G6Pk	57.0	40.3	48.5	23.0	1	0	0
**9**	G4Ea	53.9	35.5	67.6	14.6	1	0	1
**10**	C9NE	53.3	32.1	14.7	17.3	1	0	0
**11**	C8Ec	48.4	35.9	43.6	13.4	0	0	0
**12**	C2Pk	46.2	7.8	62.4	16.2	0	0	1
**13**	G10Ek	46.1	1.3	45.4	14.5	0	0	0
**14**	G19Et	45.9	39.7	67.8	18.8	0	0	1
**15**	G8Ec	45.9	38.5	49.9	23.4	0	0	0
**16**	Cali3	45.9	40.9	44.0	18.4	0	0	0
**17**	G5Ec	39.8	50.6	46.2	12.6	0	1	0
**18**	G1Pf	39.4	23.8	33.0	11.5	0	0	0
**19**	G22P	37.6	33.4	41.9	20.7	0	0	0
**20**	G13Ec	36.9	30.9	49.7	6.8	0	0	0
**21**	C11Ef	36.8	12.1	49.3	21.7	0	0	0
**22**	C13EA	36.4	27.2	43.8	3.5	0	0	0
**23**	G2Po	34.8	37.8	21.1	14.3	0	0	0
**24**	G18Pk	34.0	44.0	52.5	31.2	0	0	1
**25**	C4Pa	27.1	21.7	33.1	14.2	0	0	0
**26**	C12Pn	23.7	16.0	43.3	21.7	0	0	0
**27**	C19Ef	15.1	16.1	36.1	20.7	0	0	0
**28**	C24Pa	13.1	65.1	39.0	13.3	0	1	0
**29**	Doxorubicin	60.1	46.9	58.2	16.0			

Table 3. Scrip using Galaxy software to UPLC-MS data extraction**.**

Step 1: Input dataset (soft corals flow)
Step 2: xcms.xcmsSet<br>Extraction method for peaks detection: centWave, ppm=15 max tolerated ppm m/z deviation in consecutive scans in ppm 15.<br>peakwidth=c(5,20), min,Max peak width in seconds, 5,20<br>snthresh=3, signal/Noise threshold<br>prefilter=c(3,5000))
Step 3: xcms.group<br>xset RData file, Output dataset 'xsetRData' from step 2<br>Method to use for grouping:<br>xset<-group(xset,bw=2,mzwid=0.015,minfrac=0.3)<br>density Bandwidth: 2<br>Minimum fraction of samples necessary: 0.3<br>Width of overlapping m/z slices: 0.015<br>Maximum number of groups to identify in a single m/z slice (50)
Step 4: xcms.retcor, Method to use for retention time correction<br>xset RData file, Output dataset 'xsetRData' from step 3<br>xset2<-retcor(xset, method="obiwarp", plottype ="deviation")<br>peakgroups<br>Smooth method<br>loess<br>Number of extra peaks to allow in retention time correction correction groups: 1<br>Number of missing samples to allow in retention time correction groups: 1<br>Degree of smoothing for local polynomial regression fitting: 0.2<br>Family, gaussian, plottype, deviation.
Step 5: xcms.group, Method to use for grouping<br>xset RData file, output dataset 'xsetRData' from step 4<br>xset3<-group(xset2,bw=2,mzwid=0.015,minfrac=0.3)<br>density Bandwidth: 2<br>Minimum fraction of samples necessary: 0.3<br>Width of overlapping m/z slices: 0.015<br>Maximum number of groups to identify in a single m/z slice: 50
Step 6: xcms.fillPeaks, Filling method<br>xset RData file<br>Output dataset 'xsetRData' from step 5

xset = fillPeaks(chrom)
Step 7: CAMERA.annotate<br>RData file,<br>Output dataset 'xsetRData' from step 6<br>num_digits: 4<br>groupFWHM: multiplier of the standard deviation: 6<br>groupFWHM: percentage of FWHM width: 0.6<br>findIsotopes: max. ion charge: 3<br>findIsotopes: max. number of expected isotopes: 4<br>findIsotopes: The percentage number of samples, which must satisfy the C12/C13 rule for isotope annotation: 0.5<br>General ppm error: 5<br>Mzabs: 0.015<br>General used intensity value<br>findAdducts: Which polarity mode was used for measuring of the ms sample negative<br>groupCorr: correlation threshold (0.1), 0.75<br>groupCorr: Method selection for grouping peaks after correlation analysis into pseudospectra<br>hcs, groupCorr: significant correlation threshold (0.05)<br>findAdducts: If no ruleset is provided, calculate ruleset with max. number n of [nM+x]<br>diffreport: number of the most significantly different analytes to create EICs for, (50)<br>diffreport: width (in seconds) of EICs produced, (200)<br>diffreport: logical indicating whether the reports should be sorted by p-value, (True)
